# The Association between Serum Vitamin D Levels and Urinary Tract Infection Risk in Children: A Systematic Review and Meta-Analysis

**DOI:** 10.3390/nu15122690

**Published:** 2023-06-09

**Authors:** Yan Gan, Siyi You, Junjie Ying, Dezhi Mu

**Affiliations:** 1 Department of Pediatrics, West China Second University Hospital, Sichuan University, Chengdu 610041, China; 2 Key Laboratory of Birth Defects and Related Diseases of Women and Children (Sichuan University), Ministry of Education, NHC Key Laboratory of Chronobiology, Sichuan University, Chengdu 610041, China

**Keywords:** serum vitamin D, urinary tract infection, children, meta-analysis

## Abstract

The association between serum vitamin D levels and urinary tract infection (UTI) in children is unclear. We undertook a systematic review and meta-analysis to evaluate the relationships between different vitamin D levels and the likelihood of UTI in children. Online databases, including Web of Science, PubMed, Embase, and Cochrane Library, were searched up to 6 February 2023 for studies based on the inclusion criteria. Weighted mean difference (WMD) and Odds Ratios (ORs), along with their 95% confidence intervals (CI), were calculated, and the random-effects model was used for analysis. Twelve case–control studies and one cross-sectional study (839 children with UTI and 929 controls) were included. We found that children with UTI had lower levels of serum vitamin D than healthy controls (WMD: −7.730, 95% CI: −11.57, −3.89; *p* < 0.001). Low vitamin D levels were significantly associated with UTI in children (OR: 2.80; 95% CI: 1.55, 5.05; *p* = 0.001). The likelihood of children having a UTI significantly increased when their serum vitamin D level was less than 20 ng/mL (OR: 5.49, 95% CI: 1.12, 27.04; *p* = 0.036). Therefore, vitamin D level, especially when less than 20 ng/mL, is a risk factor in UTI.

## 1. Introduction

Urinary tract infection (UTI), including pyelonephritis and cystitis, is a common bacterial infection in children of all ages, with a prevalence ranging from 1.8% to 7.5% [1]. UTI is mainly caused by Escherichia coli (70–90%), and the other common pathogenic bacteria include Klebsiella, Proteus, Enterococcus, and Enterobacter species [2,3]. If not treated promptly, UTI may lead to renal scarring, and even severe renal disease [4]. Tc 99–labeled dimercaptosuccinic acid scanning revealed renal defects in 85% of children with febrile UTI, and permanent renal scarring occurred in 10–40% of them [5]. Therefore, timely treatment of UTI and early identification of the risk of renal damage after UTI are important, but it would be preferable if UTI could be prevented.

Vitamin D receptor (VDR) and vitamin D-activating enzyme (1α-hydroxylase) are found in many types of immune cells. Serum 25(OH)D is activated and converted into 1,25(OH)2D, the active form of vitamin D, in immune cells. 1,25(OH)2D binds to the VDR to regulate DNA transcription, stimulate the expression of antimicrobial peptides, and enhance innate immune responses [6,7,8].

Following the discovery of the VDR, increasing research has been conducted on the role of vitamin D in regulating the immune system [9,10]. Many studies have shown that vitamin D plays a protective role in innate and adaptive immunity [11,12,13]. Vitamin D deficiency has been considered to be associated with infections, mostly in adult patients but also in pediatrics, including with the recent COVID-19 pandemic and UTI [14,15,16,17,18,19,20]. Vitamin D has been found to reduce the inflammatory response by reducing interferon-γ production in the immune system, and up-regulating antimicrobial peptides in the bladder during Escherichia coli infections, thereby reducing urethral damage [17,21].

Many clinical studies have reported a relationship between serum vitamin D levels and UTI in children, demonstrating that vitamin D insufficiency or deficiency may be a risk factor for UTI [22,23,24,25,26,27,28,29,30,31,32]. Vitamin D supplementation may prevent UTI [33]. However, some studies have shown no significant relationship between serum vitamin D levels and UTI in childhood, and vitamin D supplementation may even increase the risk of infection [34,35,36]. The relationship between vitamin D levels and UTI in children is controversial; a systematic review and meta-analysis has been conducted, but the included studies were limited [37]. Our aim was to update the analysis and to evaluate the relationships between different vitamin D levels and the likelihood of UTI in children.

## 2. Materials and Methods

The systematic review and meta-analysis were conducted based on the Preferred Reporting Items for Systematic Reviews and Meta-Analyses (PRISMA), which was registered with the International Prospective Register of Systematic Reviews (PROSPERO) (no. CRD42023398469).

### 2.1. Search Strategy

The literature search was independently conducted by two researchers using the Web of Science, PubMed, Embase, and Cochrane Library databases for articles published up to 6 February 2023. In our search, we used a combination of mesh terms and their free forms.

### 2.2. Selection Criteria

The inclusion criteria were based on PICOS as follows: population (P), children (aged < 18 y); intervention/exposure (I), serum vitamin D levels; comparison (C), vitamin D sufficiency; outcome (O), UTI; and type of study (S), cohort studies/case–control studies/cross-sectional studies. The exclusion criteria were non-English articles, repeatedly published articles, articles with incomplete data, and those without control groups. Based on the inclusion and exclusion criteria, the publications were reviewed and screened independently by two authors, who sought help from a third author to resolve any disagreements.

### 2.3. Data Extraction

The following information was collected from the available articles: title of the study; first author’s name; publication year; study design; research location (country); sample size; participants’ age; the timing of serum vitamin D level evaluation (before, during, or after onset of UTI); laboratory test methods for serum 25(OH) D levels (including chemiluminescent immunoassay, ELISA, radioimmunoassays, liquid chromatography, and enzyme immunoassays); definition of vitamin D deficiency, insufficiency, or sufficiency (The Endocrine Society guidelines defined serum vitamin D levels below 20 ng/mL as deficient, 20 to 30 ng/mL as insufficient, and above 30 ng/mL as sufficient [38]); mean ± standard deviation (SD) of serum vitamin D levels in children with UTI and controls; the frequency of children with vitamin D deficiency, insufficiency, or sufficiency in both groups with or without UTI; and effect estimation (e.g., Odds Ratio [OR], Relative Risk [RR], or adjusted OR/RR) for UTI in children with vitamin D deficiency or insufficiency compared with vitamin D sufficient children. Two authors screened the data independently and sought help from a third author to resolve any disagreements.

### 2.4. Quality Assessment

The quality of the included studies was evaluated independently by two authors using the Newcastle-Ottawa Scale (NOS). This scale assesses three major aspects: selection (four items), comparability (one item), and exposure or outcome (three items). Each study had a maximum of one star per item for “selection” and “exposure or outcome”, but a maximum of two stars per item for “comparability”. Therefore, the highest NOS score in the present study was 9. In this study, scores of 0–3, 4–6, and 7–9 were considered low, moderate, and high quality, respectively.

### 2.5. Statistical Analysis

The weighted mean difference (WMD) and 95% confidence interval (CI) were used to assess serum vitamin D levels in children with UTI compared with controls. If the concentration of serum vitamin D was presented in nmol/L, it was converted to ng/mL using the SI units Conversion Calculator (https://unitslab.com/node/84 (accessed on 11 February 2023)).

Several studies have reported the frequency of children with deficient, insufficient, or sufficient vitamin D in UTI and non-UTI groups. In addition, some studies reported adjusted OR and 95% CI. Therefore, we conducted a meta-analysis of studies with data on the frequency and adjusted ORs.

A random-effects model was used for analysis. We quantified the statistical heterogeneity between studies using the chi-square test and the *I*^2^ statistics. When *I*^2^ ≥ 50%, it was considered to indicate substantial heterogeneity.

Subgroup analyses based on different continents, research quality, and methods of serum vitamin D measurement were conducted to explore the possible sources of heterogeneity between studies. Subgroup analyses were also performed based on sex and vitamin D levels. A funnel plot was constructed to intuitively reflect publication bias. Additionally, the Egger’s test was used to quantitatively analyze publication bias. To evaluate the robustness of the results, sensitivity analysis was conducted using a one-by-one elimination method. Next, trim-and-fill funnel plots were constructed to confirm the stability of the results. All statistical analyses were performed using STATA15.1. *p* < 0.05 was considered as statistically significant.

## 3. Results

### 3.1. Literature Search

**Figure 1:**
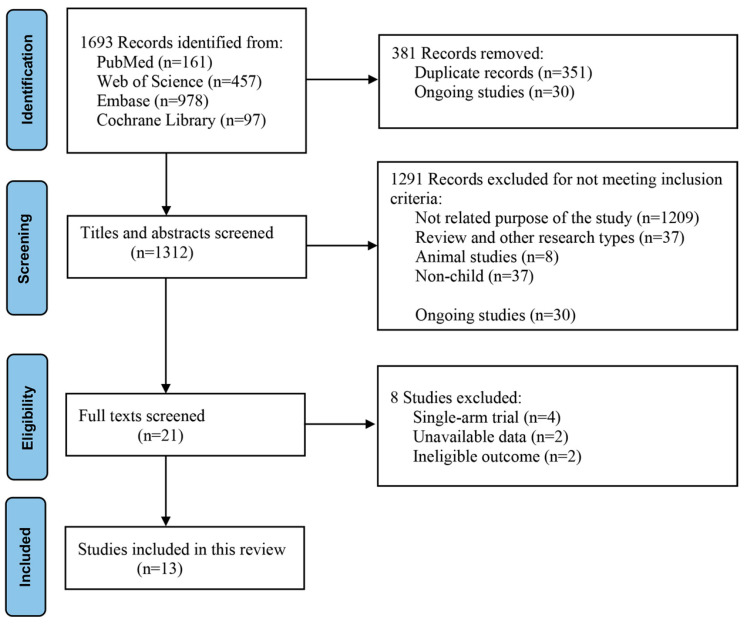
PRISMA search flowchart and studies included in the systematic review and meta-analysis.

Our search identified 1693 publications. Thirty ongoing trials, identified in the Cochrane Library, were excluded. After automatic reduction and manual screening of the publication year and title, 351 duplicates were excluded. A further 1291 articles were excluded after screening titles and abstracts. Eight studies were excluded after full-text screening. Finally, 13 studies met the eligibility criteria and were included in this systematic review and meta-analysis (Figure 1).

### 3.2. Characteristics and Quality Assessment of the Included Studies

Twelve case–control studies and one cross-sectional study were included, reporting on 839 children with UTI and 929 controls, aged 3 months to 12 years. Five studies were conducted in Iran [23,27,28,34,35], three in Turkey [22,25,39], and one each in China [33], South India [24], Romania [30], Egypt [29], and Sweden [26]. Serum 25(OH)D or 25(OH)D3, as markers for vitamin D levels, were measured using chemiluminescent immunoassay [24,25,26,27,30], ELISA [22,23,28,39], radioimmunoassays [33,35], liquid chromatography [34], and enzyme immunoassays [29]. Serum vitamin D levels were evaluated after the patients were diagnosed with a UTI while hospitalized [22,23,24,25,26,27,28,29,30,33,34,35,39]. Different criteria were used to define deficient, insufficient, and sufficient levels of serum vitamin D. For example, several studies defined vitamin D deficiency as less than 20 ng/mL [23,24,25,26,27,28,30,33,35,39]. However, two studies defined it as less than 10 ng/mL or 12 ng/mL [22,29], whereas another study did not define it [34]. Logistic regression analysis was used in some studies to adjust for gender, age, body weight, feeding style, and sunshine duration as major confounding factors [24,28,33]. The other two studies adjusted for white blood cell count and levels of C-reactive protein as confounding variables [25,29]. However, other studies did not calculate OR or adjusted OR (Table 1).

NOS scores of the studies ranged from five to eight. Two studies were considered moderate quality, whereas the others were high quality (Table 1).

### 3.3. Meta-Analyses

#### 3.3.1. Meta-Analysis of Serum Vitamin D Levels

**Figure 2:**
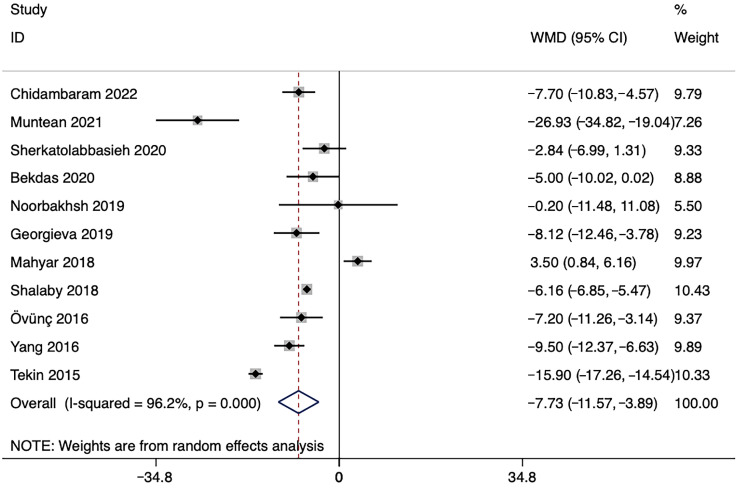
Forest plot showing the WMD in serum vitamin D levels between children with UTI and controls. The results illustrated that vitamin D levels in children with UTI were on average 7.73 ng/mL lower than those in the controls. Abbreviations: UTI, urinary tract infection; WMD, weighted mean difference [22,23,24,25,26,29,30,33,34,35,39].

Overall, 11 of the included studies reported mean ± SD in serum vitamin D levels in children with or without UTI. The meta-analysis showed that serum vitamin D level was 7.730 ng/mL lower in children with UTI than that in controls (95% CI: −11.57, −3.89 ng/mL; *p* < 0.001). However, significant heterogeneity was observed among the studies (*p* < 0.001; *I*^2^ = 96.2%) (Figure 2).

To explore the possible sources of heterogeneity, a subgroup analysis was performed according to factors that might influence the results. The results of subgroup analysis based on children from different regions showed significantly lower levels of vitamin D in children from Asia with UTI [24,33] (WMD: −8.68, 95% CI: −10.79, −6.56; *p* < 0.001; *I*^2^ = 0.0%). However, there were no significant differences in vitamin D levels in children with or without UTI from Europe [26,30] (WMD: −17.22, 95% CI: −35.65, 1.20; *p* = 0.067; *I*^2^ = 94.0%) or the Middle East [22,23,25,29,34,35,39] (WMD: −5.17, 95% CI: −10.41, 0.06; *p* = 0.053; *I*^2^ = 97.5%) (Figure S1A).

Based on the quality of the included studies, the subgroup analysis showed that the serum vitamin D levels in children with UTI were significantly lower in middle quality studies compared with those of the controls [26,39] (WMD: −6.79, 95% CI: −10.07, −3.50; *p* < 0.001; *I*^2^ = 0) and high quality studies [22,23,24,25,29,30,33,34,35] (WMD: −7.99, 95% CI: −12.40, −3.59; *p* < 0.001; *I*^2^ = 96.9%) (Figure S1B).

Subgroup analysis based on different methods of measuring vitamin D levels revealed that the difference was significant in studies using a chemiluminescence immunoassay [24,25,26,30] (WMD: −13.89, 95% CI: −20.04, −7.75; *p* < 0.001; *I*^2^ = 92.5%). However, there was no significant difference in studies using ELISA [22,23,39] (WMD: −2.74, 95% CI: −10.11, 4.64; *p* = 0.46; *I*^2^ = 91.0%) or radioimmunoassay [33,35] (WMD: −6.51, 95% CI: −15.02, 2.00; *p* = 0.13; *I*^2^ = 59.2%) (Figure S1C).

Sensitivity analyses were used to determine whether the heterogeneity originated from one or multiple studies. Heterogeneity was acceptable when four studies were eliminated [23,25,30,34] (*p* = 0.252, *I*^2^ = 23.3%). There was no significant heterogeneity (*p* = 0.494, *I*^2^ = 0.0%) when five studies were excluded [23,25,29,30,34]. When the above four or five studies were removed, the differences in vitamin D levels between the UTI group and controls remained significant (WMD: −6.92, 95% CI: −8.17, −5.67; *p* < 0.001; WMD: −7.81, 95% CI: −9.42, −6.20; *p* < 0.001).

Sensitivity analyses demonstrated that the effect value remained significant after removing each study.

**Figure 3:**
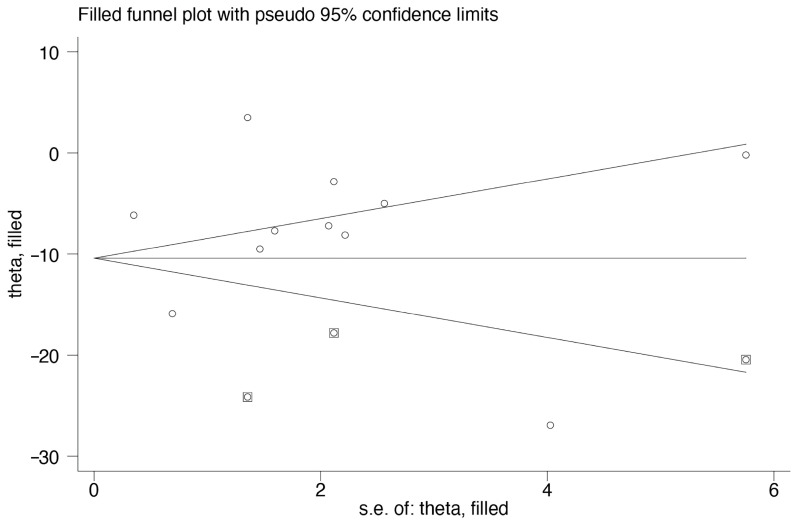
Funnel plot after trim-and-fill method in serum vitamin D levels between children with UTI and controls. Abbreviations: UTI, urinary tract infection. The circle indicates the outcome of each included study, while the square indicates the imputed studies so as to generate symmetrical funnel plots.

Slight asymmetry was observed in the funnel plots. However, the difference was still significant in the trim-and-fill analysis (WMD: −10.41, 95% CI: −14.43, −6.39; *p* < 0.001) (Figure 3). Moreover, Egger’s test revealed no significant publication bias (*p* = 0.918).

#### 3.3.2. Meta-Analysis of ORs

**Figure 4:**
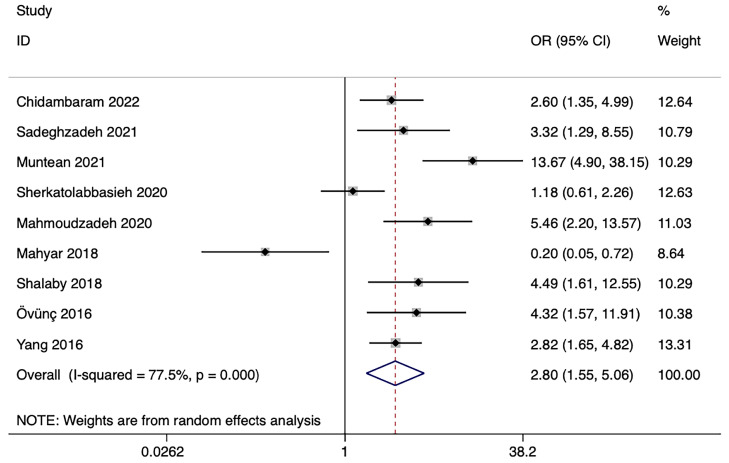
Forest plots illustrating the association between serum vitamin D levels and UTI in children. ORs were calculated from the frequency of exposure in the UTI and control groups. Exposure means low levels of serum vitamin D levels (including: insufficiency and deficiency). Abbreviations: UTI, urinary tract infection; ORs, Odds Ratios [22,23,24,27,28,29,30,33,34].

From the nine studies, we derived the frequency of sufficient and low vitamin D levels (including insufficiency and deficiency) in children with UTI and controls. The analysis of ORs demonstrated a significant association between UTI and low vitamin D levels (OR: 2.80; 95% CI: 1.55, 5.06; *p* = 0.001). Heterogeneity was significant between the studies (*p* < 0.001; *I*^2^ = 77.5%) (Figure 4).

**Figure 5:**
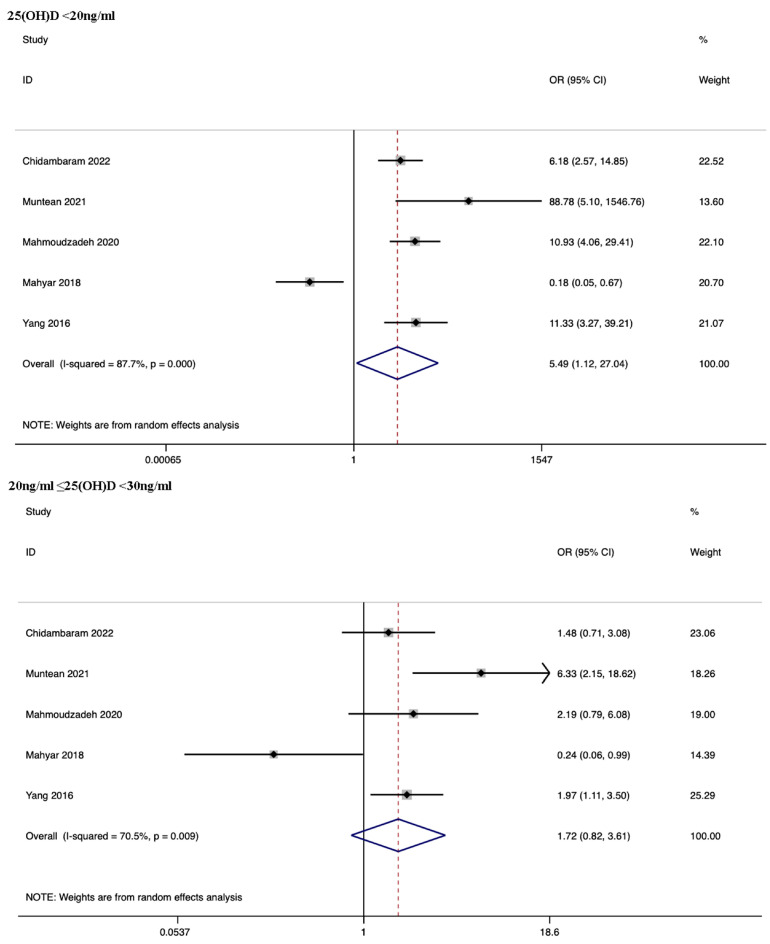
Subgroup analysis—forest plots of likelihood of UTI in children based on vitamin D levels. ORs were obtained from the frequency of vitamin D levels less than 20 ng/mL, between 20 and 30 ng/mL, and more than 30 ng/mL. Abbreviation: UTI, urinary tract infection [23,24,27,30,33].

We carried out a subgroup analysis based on different levels of serum vitamin D, and the results showed that children were significantly more likely to develop UTI when their serum vitamin D levels were less than 20 ng/mL (OR: 5.49, 95% CI: 1.12, 27.04; *p* = 0.036; *I*^2^ = 87.7%). However, there was not a significant association between vitamin D levels and the likelihood of UTI when serum vitamin D levels were between 20 and 30 ng/mL (OR: 1.72, 95% CI: 0.82, 3.61; *p* = 0.150; *I*^2^ = 70.5%) (Figure 5).

We also carried out a subgroup analysis based on sex for two studies that stratified by sex [22,28]. The results showed a significant association between low vitamin D levels and the likelihood of UTI in female children (OR: 5.00, 95% CI: 2.19, 11.39; *p* < 0.001; *I*^2^ = 0.0%), but not in male children (OR: 1.43, 95% CI: 0.37, 5.55; *p* = 0.604; *I*^2^ = 0.0%) (Figure S2).

The results of the subgroup analysis based on children from different regions showed a significant association between low vitamin D levels and the likelihood of UTI in the studies conducted in Asia [24,33] (OR: 2.73, 95% CI: 1.80, 4.13; *p* < 0.001; *I*^2^ = 0.0%) and in Europe [30] (OR: 13.67, 95% CI: 4.90, 38.15; *p* < 0.001; *I*^2^ = 0.0%). However, there was not a significant association for the studies conducted in the Middle East [22,23,27,28,29,34] (OR: 2.18, 95% CI: 0.92, 5.14; *p* = 0.076; *I*^2^ = 79.9%) (Figure S3).

Sensitivity analysis showed significant differences after the removal of each study. Egger’s test revealed no significant publication bias (*p* = 0.296).

#### 3.3.3. Meta-Analysis of Adjusted ORs

**Figure 6:**
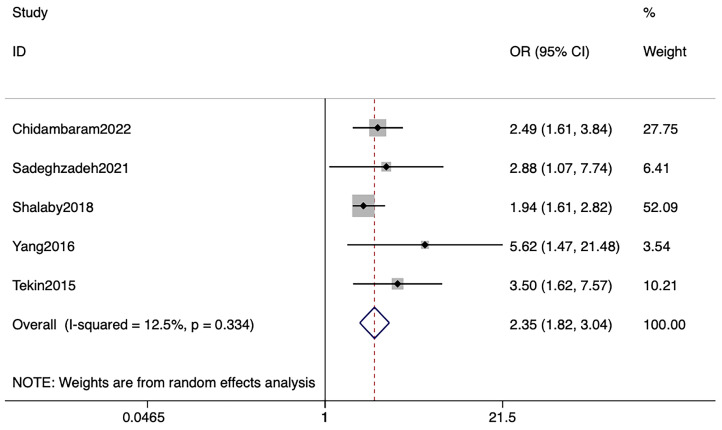
Forest plots illustrating the association between serum vitamin D levels and UTI in children. Adjusted ORs were reported by five studies. Abbreviations: UTI, urinary tract infection; ORs, Odds Ratios [24,25,28,29,33].

Adjusted OR values in five studies were separately extracted for meta-analysis [24,25,28,29,33], and the results showed that children with vitamin D deficiency (four studies defined vitamin D deficiency as less than 20 ng/mL and one study defined it as less than 10 ng/mL) have a 2.35 times greater chance of developing UTI than those with vitamin D sufficiency (OR: 2.35; 95% CI: 1.82, 3.04; *p* < 0.001) with acceptable heterogeneity (*p* = 0.334; *I*^2^ = 12.5%) (Figure 6).

The results of subgroup analysis based on different regions showed a significant association between vitamin D deficiency and the likelihood of UTI in the studies conducted in Asia [24,33] (OR: 2.89, 95% CI: 1.55, 5.37; *p* = 0.001; *I*^2^ = 22.2%) and the Middle East (OR: 2.24, 95% CI: 1.59, 3.15; *p* < 0.001; *I*^2^ = 16.3%) (Figure S4).

Sensitivity analysis showed that the overall effect remained significant after removing each study.

## 4. Discussion

A meta-analysis of serum vitamin D levels found that children with UTI had lower serum vitamin D levels than healthy controls. A meta-analysis of ORs showed a significant association between low vitamin D levels and risk of UTI in children. Moreover, sensitivity analysis showed that this association was stable.

A previous systematic review and meta-analysis reported the association of serum vitamin D levels and UTI risk in children [37]. That review included six studies (UTI: 339, control: 306), whereas our review included 13 studies (UTI: 839, control: 929). Similar to our results, there was high heterogeneity among the included studies, and it was found that children with UTI had lower vitamin D levels than healthy controls. However, the earlier review found a potential publication bias, while the Egger’s test revealed no significant differences in our results. We found that the results were still statistically different when constructing trim-and-fill and sensitivity analyses. In addition, we conducted an analysis using ORs to evaluate the relationship between different vitamin D levels and the likelihood of UTI and found that there was a 5.49 times greater chance of children developing UTI when serum vitamin D levels were less than 20 ng/mL than for those with vitamin D sufficiency.

When subgroup analyses were conducted based on different regions, the results showed that in Asia, children with UTIs had lower vitamin D concentrations than healthy controls and that there was a significant association between low vitamin D levels and the risk of UTIs. However, this was not the case in Europe or the Middle East. Latitudes and regions can affect serum vitamin D levels because of sunlight exposure, which is the primary source of vitamin D for the human body. The farther away from the equator, the less sunlight a region receives, resulting in lower serum vitamin D levels for residents. However, serum vitamin D levels have been observed to be higher in northern Europe than in southern Europe, with this being the influence of diet and skin color [40,41]. In the two European studies conducted in Romania and Switzerland, local eating habits may have affected vitamin D levels. Additionally, the limited literature may not reflect the true effect value. Among studies conducted in the Middle East, one study found the opposite, showing that children with UTI had higher concentrations of vitamin D than healthy controls. When pregnant women have vitamin D deficiency, their babies also develop vitamin D deficiency. In the Middle East, women have extremely low levels of serum vitamin D due to skin-covering clothes and lack of sun exposure [42,43]. Furthermore, serum vitamin D levels show high variability among countries in the Middle East [40], which may be a factor in the Middle East subgroup analysis results that is inconsistent with the overall effect. Low vitamin D levels have been observed in Asian countries such as China, Mongolia, and India, while levels are higher in Japan and Malaysia [40,44,45,46,47]. The two studies in Asia included in our meta-analysis were conducted in China and India, and the subgroup results showed that children with UTI had lower serum vitamin D levels than controls, and a low vitamin D level was strongly associated with the likelihood of UTI. Subgroup analysis of the adjusted OR based on regions showed that children with vitamin D deficiency have a 2.89 times greater chance of developing UTI than healthy controls in Asia, compared with 2.35 times in the Middle East. This may be attributed to a variety of factors affecting UTI and not just serum vitamin D levels.

Two studies conducted a stratified analysis according to the sex of the children, and we conducted a meta-analysis of the ORs for boys and girls. The results showed a significant association between low vitamin D levels and the likelihood of UTI in female children, but not in male children. Due to the structure of the urinary tract, females are more likely to develop UTI than males, which may explain our results. However, as only two studies were included, our findings may not accurately reflect the real effects.

Subgroup analyses were also performed based on study quality and vitamin D measurement methods to identify the sources of heterogeneity. The heterogeneity did not decrease significantly. Moreover, through sensitivity analysis, we identified four studies that may be major contributors to heterogeneity [23,25,30,34]. This difference remained significant when the above four studies were excluded from the analysis. Overall, heterogeneity may be due to sex, age, ethnicity, continence, latitude, dietary habits, skin-covering clothes, cutoff value, and measurement methods for serum vitamin D.

Several points should be considered when interpreting the results of this systematic review and meta-analysis. First, the results showed that low vitamin D levels were associated with UTI; however, the included studies were case–control or cross-sectional studies, and vitamin D concentrations were measured after the children had acute UTIs, making it difficult to infer causality. Vitamin D deficiency is likely a consequence or cause of chronic inflammation. Some researchers believe that low 25(OH)D levels are not a cause, but a result of chronic inflammation [7]. However, only participants with non-recurrent UTI were included in this study. Further prospective studies are required to better explain this association. Importantly, for children with UTI, can vitamin D supplementation reduce the incidence of UTI? How vitamin D is supplemented and how much is taken is something we should be concerned about. Based on the guidelines, 400 IU of vitamin D per day was recommended for infants within 6 months, 400 to 600 IU/day for infants aged 6–12 months, and 600 IU/day for children aged 1–3 years [48]. For children older than 3 years, sunbathing was recommended, and vitamin D supplementation is not necessary [48]. Second, the studies only included Europe, Asia, and the Middle East, and the results may not be representative. Third, most studies matched season and age, while some adjusted for sex and age as confounding factors. However, other variables such as race, skin color, lifestyle, diet, and exposure to sunshine can affect vitamin D levels and may affect the overall effect values. Fourth, the cut-off values for vitamin D deficiency or insufficiency were inconsistent across studies. Most studies defined 25(OH) D levels less than 20 ng/mL as deficient, and more than 20 ng/mL and less than 30 ng/mL as insufficient [23,24,25,26,27,28,30,33,35,39]; other studies used other thresholds, which will affect the frequency of exposure. This may have been a source of heterogeneity. Finally, the meta-analysis revealed a significant heterogeneity. Although different subgroup analyses were conducted, the source of the heterogeneity could not be determined. However, sensitivity analyses showed robust results.

## 5. Conclusions

In conclusion, low serum vitamin D levels were associated with a higher risk of UTI, especially when vitamin D levels are below 20 ng/mL. Furthermore, Asian or female children with low serum vitamin D levels may be at a greater risk of developing UTI.

## Figures and Tables

**Table 1:** Characteristics of the 13 included studies.

Study	Study Design	Country	Total Number Cases/Controls	Age	Vitamin D (ng/mL)		Exposure Number Cases/ Controls	Estimated OR (95% CI)	Newcastle -Ottawa Score
					UTI	Control			
Chidambaram 2022 [24]	case–control	South India	82/82	UTI: 2.36 ± 1.42 y control: 2.57 ± 1.26 y	24.27 ± 9.70	31.97 ± 10.7	60/42	Adjusted OR: 2.486 (1.610, 3.838)	8
Sadeghzadeh 2021 [28]	case–control	Iran	40/40	1–12 y	_	_	30/19	Adjusted OR: 2.884 (1.075, 7.738)	7
Muntean 2021 [30]	case–control	Romania	59/42	UTI: 3.95 ± 2.94 y control: 3.25 ± 3.03 y	26.06 ± 14.25	52.99 ± 23.16	41/6	_	7
Sherkatolabbasieh 2020 [34]	case–control	Iran	44/214	UTI: 6.58 ± 1.57 y control: 6.7 ± 1.58 y	29.2 ± 12.27	32.04 ± 15.04	25/113	_	8
Mahmoudzadeh 2020 [27]	case–control	Iran	75/75	UTI: 6.9 ± 4.2 y control: 7.9 ± 3.8 y	_	_	68/48	_	7
Bekdas 2020 [39]	case–control	Turkey	43/24	UTI: 6.9 ± 4.2 y control: 7.9 ± 3.8 y	18 ± 9	23 ± 10.6	_	_	6
Noorbakhsh 2019 [35]	case–control	Iran	25/40	mean: 2.7 y	45.7 ± 21.05	45.9 ± 24.8	_	_	7
Georgieva 2019 [26]	cross-section	Sweden	76/44	4.5–33.5 m	32.32 ± 8.48	40.44 ± 13.2	_	_	5
Mahyar 2018 [23]	case–control	Iran	70/70	UTI: 53.2 ± 35.6 m control: 62.2 ± 36.1 m	20.4 ± 8.6	16.9 ± 7.4	57/67	_	8
Shalaby 2018 [29]	case–control	Egypt	50/50	UTI: 0.98 ± 1.15 y control: 0.90 ± 1.23 y	4.2 ± 1.08	10.36 ± 2.24	19/6	Adjusted OR: 1.94 (1.61, 2.82)	7
Övünç 2016 [22]	case–control	Turkey	36/38	UTI: 6.8 ± 3.6 y control: 6.3 ± 2.8 y	16.5 ± 6.3	23.7 ± 11	28/17	_	7
Yang 2016 [33]	case–control	China	132/106	UTI: 7.29 ± 3.06 m control: 7.09 ± 3.25 m	29.09 ± 9.56	38.59 ± 12.41	74/33	Adjusted OR: 5.619 (1.469, 21.484)	7
Tekin 2015 [25]	case–control	Turkey	82/64	UTI: 2.57 ± 2.56 y control: 2.10 ± 1.37 y	11.7 ± 3.3	27.6 ± 4.7	_	Adjusted OR: 3.503 (1.621, 7.571)	8

Exposure: low levels of serum vitamin D (including: insufficiency and deficiency); Mean ± SD (all such values); UTI: urinary tract infection.

## Data Availability

All relevant data are within the manuscript.

## Supplementary Materials

The following supporting information can be downloaded at: https://www.mdpi.com/article/10.3390/nu15122690/s1, Figure S1: Subgroup analysis—forest plot of serum vitamin D levels in children with/without UTI. (A) Subgroup analysis based on children from different continents. (B) Subgroup analysis based on the quality of the included studies. (C) Subgroup analysis based on different measurement methods for serum vitamin D. Abbreviations: UTI, urinary tract infection. Figure S2: Subgroup analysis—forest plots of the likelihood of UTI in children based on sex. ORs were obtained from the frequency of exposure in the UTI and control groups. “Overall”: the result obtained regardless of sex. Exposure: low levels of serum vitamin D (including: insufficiency and deficiency). Abbreviations: UTI, urinary tract infection. Figure S3: Subgroup analysis—forest plots of the likelihood of UTI in children based on region. ORs were obtained from the frequency of exposure in the UTI and control groups. Exposure: low levels of serum vitamin D (including: insufficiency and deficiency). Abbreviations: UTI, urinary tract infection. Figure S4: Subgroup analysis—forest plots of adjusted ORs for the likelihood of UTI in children according to regions. Adjusted ORs were reported by five studies. Abbreviations: UTI, urinary tract infection; ORs, Odds Ratios.
