# Supplementary material for: The Association between Serum Vitamin D Levels and Urinary Tract Infection Risk in Children: A Systematic Review and Meta-Analysis
Source: Nutrients. 2023 Jun 9;15(12):2690. doi: 10.3390/nu15122690 (PMC10305600; doi:10.3390/nu15122690)
Supplement: Supplementary file 1 [file nutrients-15-02690-s001.zip › nutrients-2423539-supplementary.pdf]

Supplementary Materials

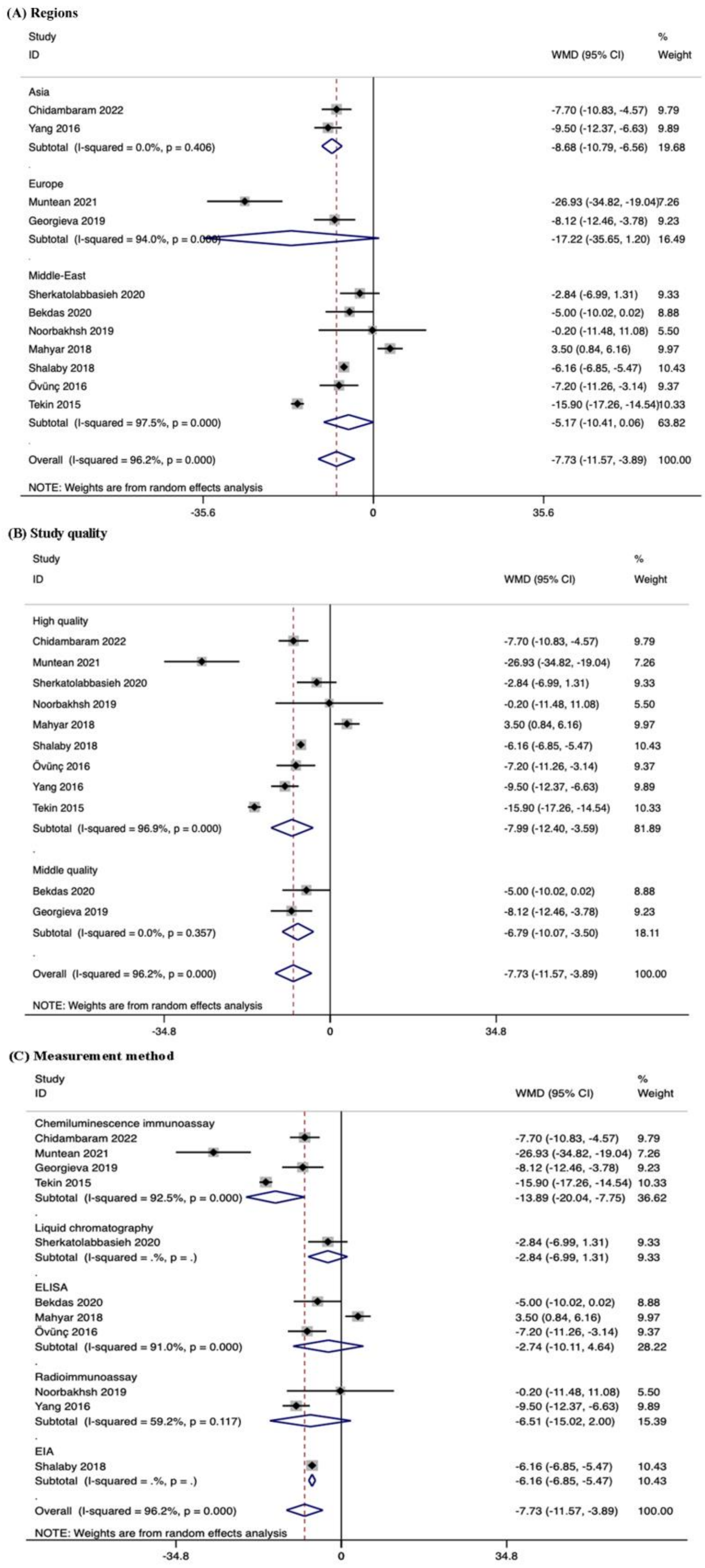

Figure S1. Subgroup analysis-forest plot of serum vitamin D levels in children with/without UTI. (A) Subgroup analysis based on children from different continents. (B) Subgroup analysis based on the quality of included studies. (C) Subgroup analysis based on different measurement methods for serum vitamin D. Abbreviations: UTI, urinary tract infection.

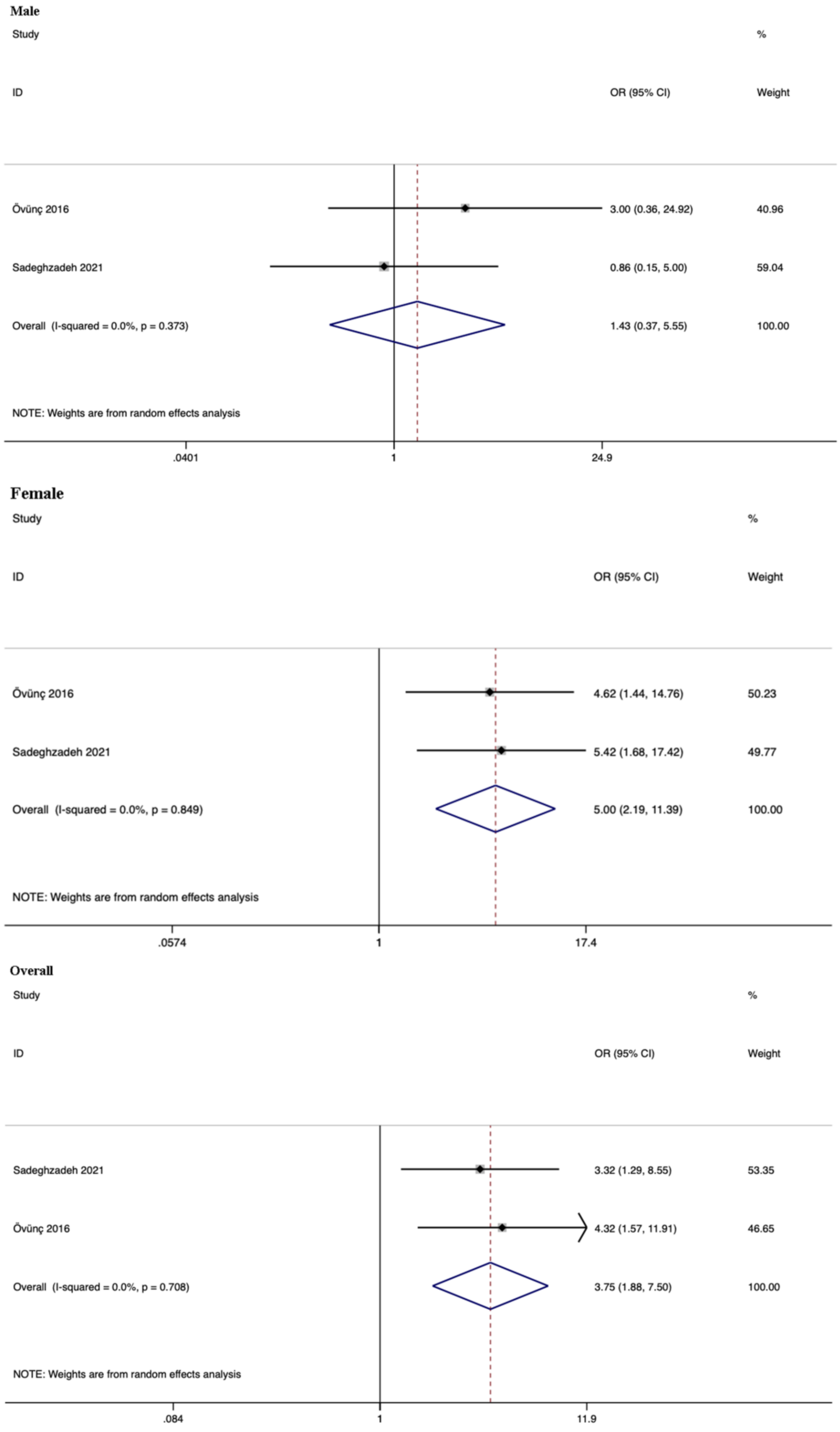

Figure S2. Subgroup analysis-forest plots of likelihood of UTI in children based on sex. ORs were obtained from the frequency of exposure in the UTI and control groups. "Overall": the result obtained regardless of sex. Exposure: low levels of serum vitamin D (including: insufficiency and deficiency). Abbreviation: UTI, urinary tract infection.

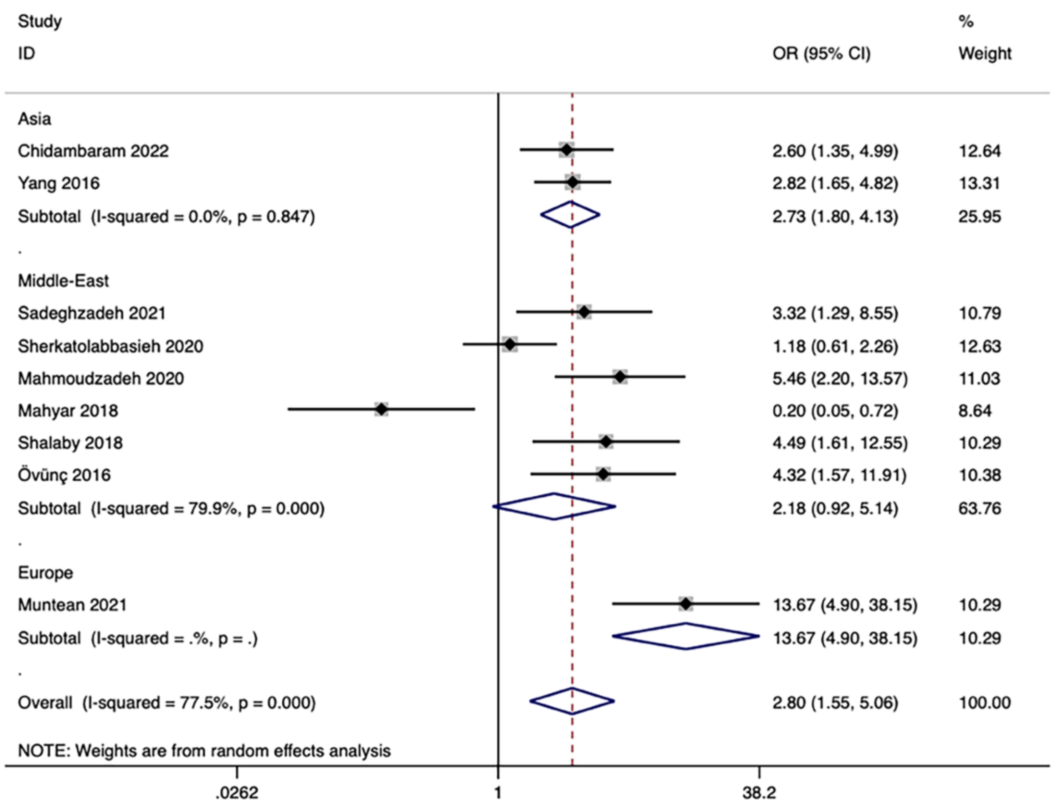

Figure S3. Subgroup analysis-forest plots of likelihood of UTI in children based on regions. ORs were obtained from the frequency of exposure in the UTI and control groups. exposure: low levels of serum vitamin D (including: insufficiency and deficiency). Abbreviations: UTI, urinary tract infection.

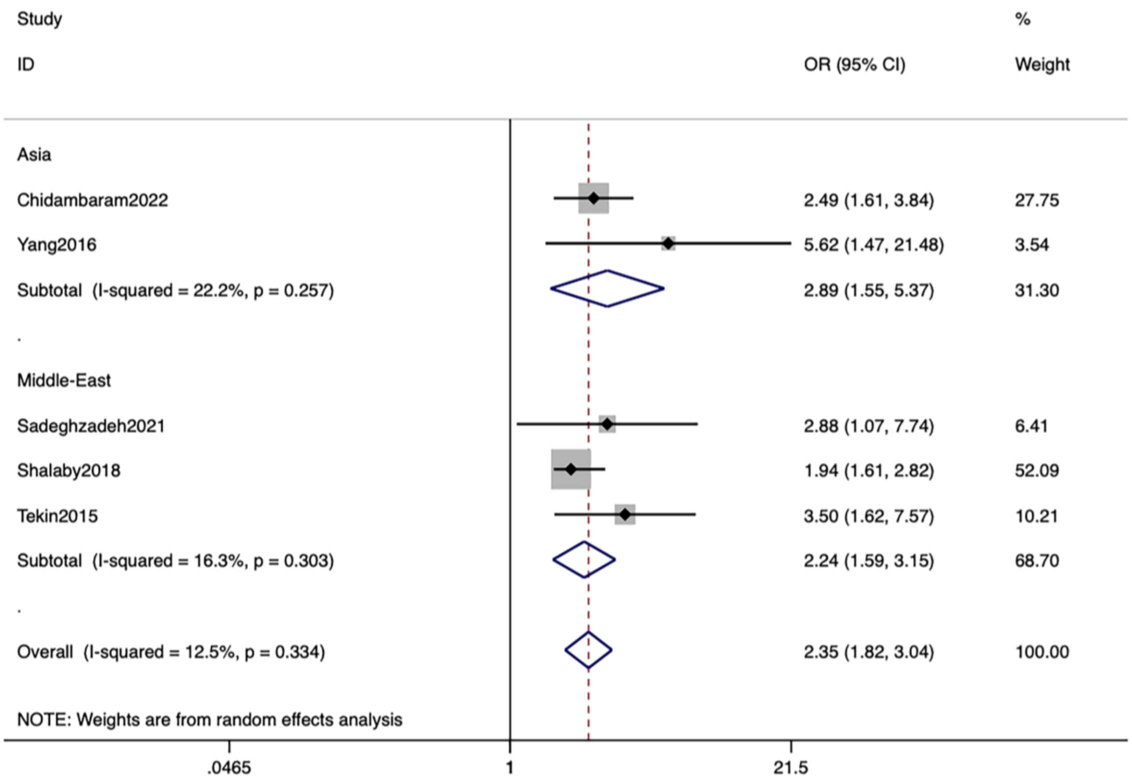

Figure S4. Subgroup analysis-forest plots of adjusted ORs for likelihood of UTI in children according to regions. Adjusted ORs were reported by five studies. Abbreviations: UTI, urinary tract infection; ORs, Odds Ratios.
